# On The Relationship between Suspended Solids of Different Size, the Observed Boundary Flux and Rejection Values for Membranes Treating a Civil Wastewater Stream

**DOI:** 10.3390/membranes4030414

**Published:** 2014-08-04

**Authors:** Marco Stoller, Javier Miguel Ochando Pulido, Luca Di Palma

**Affiliations:** 1 Department of Chemical Materials Environmental Engineering, University of Rome “La Sapienza”, Via Eudossiana 18, Rome 00184, Italy; E-Mail: luca.dipalma@uniroma1.it; 2 Chemical Engineering Department, University of Granada, Granada 18071, Spain; E-Mail: jmochandop@ugr.es

**Keywords:** membrane fouling, membrane selectivity, boundary flux, wastewater treatment

## Abstract

Membrane fouling is one of the main issues in membrane processes, leading to a progressive decrease of permeability. High fouling rates strongly reduce the productivity of the membrane plant, and negatively affect the surviving rate of the membrane modules, especially when real wastewater is treated. On the other hand, since selectivity must meet certain target requirements, fouling may lead to unexpected selectivity improvements due to the formation of an additional superficial layer formed of foulants and that act like a selective secondary membrane layer. In this case, a certain amount of fouling may be profitable to the point where selectivity targets were reached and productivity is not significantly affected. In this work, the secondary clarifier of a step sludge recirculation bioreactor treating municipal wastewater was replaced by a membrane unit, aiming at recovering return sludge and producing purified water. Fouling issues of such a system were checked by boundary flux measurements. A simple model for the description of the observed productivity and selectivity values as a function of membrane fouling is proposed.

## 1. Introduction

In 2011, Field and Pearce introduced a new concept of threshold flux to distinguish low fouling and high fouling operating conditions for both continuous and batch modes [1]. Moreover, it was observed that compared to the sustainable flux, which embody only economic criterions, the threshold flux is somehow connected to the critical flux at its basis level, which is the appearance of fouling phenomena. Indeed, the latter concept extends the previous theory of the critical flux, previously introduced by Field *et al*. [2] and successively merged together by Stoller *et al.* [3] in a common term, that is the boundary flux.

Many studies already showed that a decrease of the boundary flux values occur when the suspension concentration rises. Some researchers observed a rapid decrease of the boundary flux at increasing concentration of latex suspensions [4,5]. The plotting of the permeate flux as a function of the logarithm of the concentration was not linear, and the film model was not satisfied. As a consequence, it appeared that neither the film nor the gel model were capable of explaining the phenomena. This discrepancy was attributed to the variation of diffusion coefficient or viscosity and/or the presence of surface interaction between membrane surface and solutes [6]. Moreover, other studies already assessed that the effect of the particle size on the critical flux cannot be experimentally determined, since, for an accurate analysis, particles with different sizes but the same surface properties are required [7].

This appears to be a critical aspect, especially in the treatment of real wastewater, where the nature of the particles leading to fouling issues is widely heterogeneous. In the case of olive mill vegetation wastewater in contact with ultrafiltration and nanofiltration membranes, Stoller *et al.* and Ochando-Pulido *et al.* [8,9,10] observed that a quantitative parameter, such as the COD of the feedstock, was not sufficient to describe the system, since it did not distinguish different molecules having different behavior. More recent studies showed that beside quantitative measurements, a qualitative characterization of the feedstock is required to obtain information about the particle size distribution (PSD) of suspended matter at nanoscale [11,12,13]. To this aim, the use of a nanosizer has been proved to be useful, providing the number of particles belonging a certain dimensional class only by indicating a percentage compared to other classes rather than a number. In addition, it was found that particles exhibiting sizes ranging from 10 times lower to 10 times higher to that of the average pore membrane size should to be considered. By a combination of quantitative (COD) and qualitative (PSD) measurements, a reliable fingerprint of the feedstock was found and fitting of the boundary flux data was successfully performed [14]. This appears to be a critical point to permit proper membrane process design inhibiting fouling issues [15,16].

In this work, the fouling of membranes treating the effluent of a step sludge recirculation (SSR) activated sludge process treating municipal wastewater was investigated. With respect to conventional activate sludge reactors, the SSR system has been proved to achieve higher carbon and nitrogen removal with minimum sludge production, whose disposal of represents one of the crucial issues in wastewater management [17,18].

Membranes at the ultrafiltration and nanofiltration range have the additional advantage of selectively separating different microorganisms, such as bacteria and protozoa, from the biomass. This should enhance the process performances and is not possible to achieve by the use of a sedimentation tank. Boundary flux values and selectivity were investigated by using two different feed streams, one consisting in the effluent of the SSR, the other consisting on the same stream after pretreatment by ultrafiltration.

Finally, a simple model on the basis of the formation of fouling acting as a secondary membrane layer was successfully developed in order to describe the observed results and highlight the differences between the two proposed configurations.

## 2. Experimental Section

The SSR configuration is a modified version of a conventional multistage system CSTR, where the recirculation of the settled sludge from the secondary clarifier, is distributed among the stages. Some studies in the last decade have already suggested that this configuration ensures greater efficiencies both in terms of carbon and nitrogen removal rate with respect to the conventional system [19,20]. This increase of performances was attributed to the establishment of an optimal distribution of microorganisms along the reactor, thus favoring substrate degradation in the first stages and predation in the latter stages. In addition, an increase of sludge settleability was observed [17].

In this present work, a synthetic wastewater simulating a typical municipal wastewater in Italy was used. The carbon source was methanol, while nutrients were provided according to the optimal BOD5:N:P mass ratio of 100:5:1 by adding ammonium chlorine and potassium phosphate [21]. Tap water is used to prepare the influent, in order to provide the micronutrients necessary for biomass growth. The mixed liquor from an activated sludge plant was used to inoculate the reactor.

**Figure 1:**
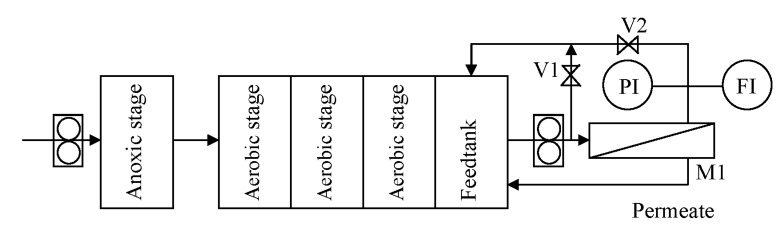
Scheme of the experimental setup.

The experimental setup is shown in Figure 1.

The SSR system consisted of one anoxic stage and three aerobic compartments, each featuring a volume of 2 L. The treated water was collected in a subsequent feedtank of 2 L and sent by a recirculation pump at 400 L·h^−1^ to the membrane system, consisting of a housing (M1), where a spiral wounded membrane module of 0.52 m^2^ can be fitted. The operating pressure *P* was regulated by acting on the valves *V*1 and *V*2, up to a maximum of 3 bars. Temperature in the feedtank was kept constant at 20 °C. The concentrate was sent back to the last stage of the SSR, whereas the permeate was withdrawn as purified water. Since this study focuses on membrane fouling, to avoid any change in the feedstock characteristics during operation, the permeate stream was recycled back to the feedtank after analysis.

It was observed that the separation of bacteria and protozoa was effective by using the NF membrane, while very limited effects were observed on the UF membrane. For this reason, the study focused on the NF membrane and in a first series of tests, hereafter called ONF, nanofiltration (NF) was directly used. In a second series of tests, a different configuration was investigated, hereafter called UFNF: ultrafiltration (UF) served as a pretreatment, followed by nanofiltration (NF), and only the UF permeate was fed.

Selected characteristics of the used membrane modules and of the wastewater are reported in Table 1. The MCWO values, provided by the supplier GE Water, were converted to pore sizes *D*_p_ by using the following relationship [22]:

*D*_p_ (nm) = 0.62 e^0.000146MCWO^ (1)

**Table 1:** Characteristics of membranes and feedstock.

Membrane	Model	MWCO (Da)	Pore Size *D*_p_ (nm)
UF	Osmonics GM	8000	2.00
NF	Osmonics DL	300	0.65
**Feedstock Parameter**	**Units**	**Value**	
COD	mg/L	810.0	
TOC	mg/L	187.5	

## 3. Results and Discussion

The COD of the effluent of the SSR, that was the feedstock to the membranes, was about 73.5 mg/L.

**Figure 2:**
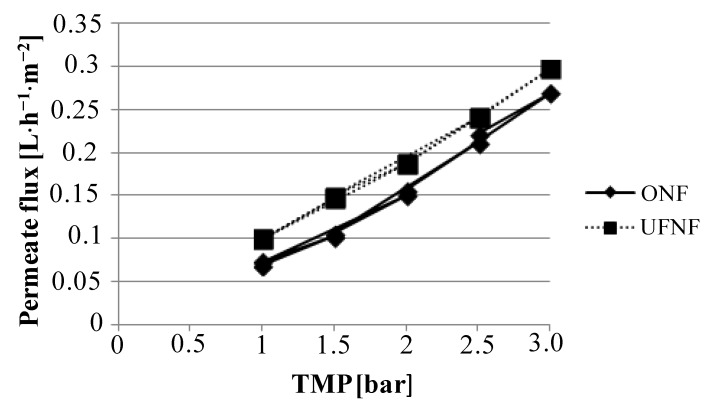
Boundary flux determination for nanofiltration (NF) membrane by using ultrafiltration (UF) NF and ONF.

For each configuration, boundary flux values were preliminarily determined by using the pressure step cycling method, described in detail elsewhere [23,24,25]. Since the membrane process supports maximum pressure values of 3 bars, boundary flux analysis was restricted to this range. The obtained results for the NF membrane are reported in Figure 2.

Osmotic pressure was found to be approximately 0.1 bar for both configurations, and as a consequence the TMP values can be considered to be the same in both experiments at same operating pressure. Boundary conditions were not found for any membrane and feedstock used within this study under the 3 bar limit of the lab plant. For this reason, an operating pressure of 2.5 bar was fixed and used for all experiments.

For COD only, a sample was withdrawn at the start of the experiment (*t* = 0). After a period of operating time T equal to 30 min, able to establish stable polarization (and reversible fouling) phenomena, separated samples of the feedstock and the permeate stream were collected. Table 2 reports the obtained permeate fluxes, together with the characteristics of the permeate stream, rejection values χ and chemical analysis.

**Table 2:** Characteristics of the feed and permeate streams for the NF membrane at *t* = *T*.

Parameter		UFNF	ONF
*J*_p_ (L·h^−1^·m^−2^)		0.2395	0.2103
COD	Feed (mg·L^−1^) at *t* = 0	23.9	73.5
	Feed (mg·L^−1^)	21.5	73.3
	Permeate (mg·L^−1^)	5.64	65.1
	χ	76.4%	11.4%
Cl^−^	Feed (mg·L^−1^)	1.1607	0.8724
	Permeate (mg·L^−1^)	0.7016	0.9140
	χ	39.5%	−4.8%
SO_4_^2−^	Feed (mg·L^−1^)	0.2091	1.4380
	Permeate (mg·L^−1^)	0.0558	1.2353
	χ	73.3%	14.1%

It is interesting to notice how selectivity was significantly affected by the pretreatment of UF: COD rejection resulted increased from 11.4% up to 76.4%. This result was unexpected, since by applying a simple sieving concept on the membranes, NF should always hold all particles being equal or bigger than the pore size. As a consequence, it would appear logical that the rejection of ONF should be higher than UFNF, where UF separates a certain amount of the particles beforehand. Conversely, in the present work, it appears not to be the case. In fact, the only explanation to the experimental results is that neither UF nor NF are capable of rejecting most of the particles, which was much smaller than the NF pore size, but using UFNF as a feedstock these particles were trapped and removed from the permeate stream.

The negative rejection value of Cl is given by a near to zero rejection value of the membrane and electrostatic phenomena on the anions, since the used membranes are slightly negatively charged [26,27].

In order to justify these results, the effect on membrane fouling on the membrane selectivity was considered.

**Figure 3:**
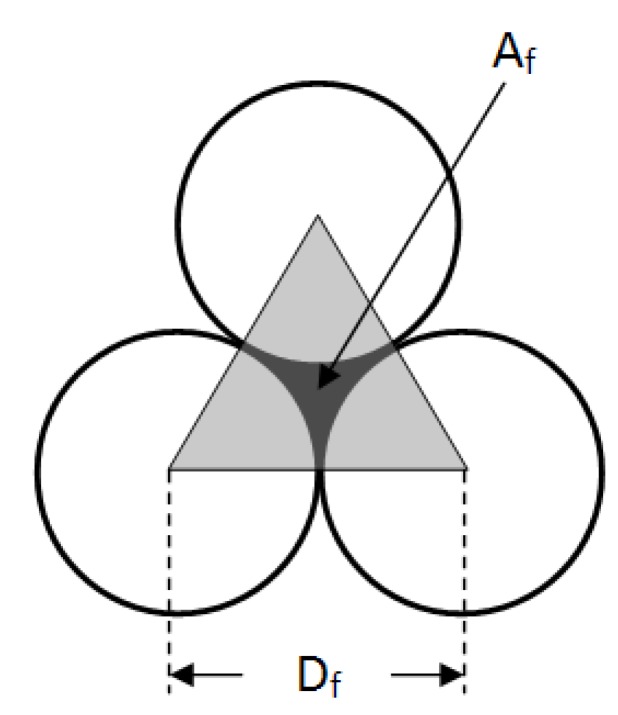
Space between particles forming the fouling layer.

Assuming operation at sub boundary flux conditions, in the presence of a mono dispersed slurry, with the hypothesis of spherical particles packing perfectly on top of the membrane surface, without phase changes, cohesion and gelification, the single particle cluster is the one shown in Figure 3.

Both the hypotheses may apply to keep the model as simple as possible, since the focus will stay on organic matter agglomerates at nanoscale, ranging in a small and narrow range from 1 nm to 6.5 nm.

Due to the low COD values and in the absence of long chained organic molecules (beside those of the biomass), sensible Donnan and/or other electrostatic adsorption and/or repulsion effects between the biomass and the membranes were neglected.

The black zone between the molecules is the minimum possible void area *A*_f_, which may be calculated by subtracting from the triangle area *A*_T_ the total area of the three smaller grey surfaces *A*_S_. A smaller particle *d*_f_ can pass through the bigger particles *D*_f_ of volume *V*_f_ and the area *A*_f_. By simple geometrical calculations on the triangle and the center point of the void, it is possible to evaluate the void area *A*_f_ and the apparent pore size *d*_f_ of the fouling layer, and as a consequence, the void space of the 2D cluster ε_f_* by the following relationships:

*d*_f_ = 0.154*D*_f_ (2)

*A*_f_ = *A*_T_ − *A*_S_ = 0.433*D*_f_^2^ − 0.392*D*_f_^2^ = 0.041*D*_f_^2^ (3)

ε_f_* = *A*_f_·*A*_T_^−1^ = 0.094 (4)

The value of ε_f_* is independent from the particle size and it assumes a constant value for the considered 2D cluster and as a consequence, for the top layer. Therefore ε_f_* is equal to the pore density of the secondary membrane, and must not be confused with the void space in the packed fouling layer ε_f_.

On the NF membrane, the fouling layer is formed by particles having a dimension up to 10 times the pore size [11,12,13]. Bigger particles than this size are affected by low interaction and have good possibility of raising from the fouling layer to the bulk, and consequently, in the concentrate stream, and can therefore be neglected. Smaller particles integrate in the fouling layer within the void spaces left by bigger particles; thus, the analysis can concentrate on the maximum dimension of particles forming the fouling layer. The biggest particles in UFNF forming the fouling layer is 2 nm (that is the cut-size of the pretreatment process, in detail the cut-size of the adopted UF membrane), whereas in the case of ONF a sum of 6.5 nm (10 times the NF pore size, considered to interfere with the membrane as fouling particles) and 2 nm particles (the same found after the UF membrane) must be considered.

Assuming the COD as representative of the amount of organic matter in the feedstock, from the data of Table 3, it is possible to calculate the mass fraction of organics in contact with the NF membrane by feeding UFNF compared to ONF, equal to 100%, by this Equation:

*m*_f_ = COD_UFNF_·COD_ONF_^−1^ (5)

For the void fraction ε_f_, this can be calculated as:

ε_f_ = (*D*_f_^3^ − *V*_f_)·*D*_f_^−3^ (6)

where *n*_f_ is the number of particles in a reference volume *V*_ref_. As expected, the value of ε_f_ decreases (although slightly) as a function of decreasing particle size.

Table 3 shows the obtained results in terms of the apparent pore size *d*_f_.

**Table 3:** Obtained results of the fouling layer characterization.

Feedstock	*D*_f_ (nm)	*V*_f_ (nm^3^)	*m*_f_ (%)	*n*_f_ (# nm^−3^)	*d*_f_ (nm)	ε_f_ (–)	COD_F_ (mg·L^−1^)
UFNF	2.0	4.19	33	0.1250	0.308	0.4762	2.4
ONF	6.5	143.72	67	0.0036	1.001	0.4766	0.2

It is interesting to notice that UFNF was capable of improving the cut-size of the NF membrane, from 0.65 nm down to 0.3 nm, whereas ONF did not lead to the same effect. The reduction of the apparent pore size, and the corresponding the reduction of the cut-size of the membrane, appears to be the reason of the sensible increase of the selectivity of the NF membrane if put in contact with the feedstock UFNF.

Finally, considering a single cluster, the thickness *x* of the fouling layer may be calculated by the Equation (7), considering the number of single overlapping layers *N*, each of height *H* and formed by *N*_L_ particles, if the membrane surface *A*_mem_ is uniformly covered:

*x* = *N*·*N*_L_^−1^·*H* = (k·*m*_f_·COD_F_)·(*D*_f_^−2^·*A*_mem_)·(0.866*D*_f_) (7)

where k is a constant, equivalent to the number of particles giving rise to the unity of COD and COD_F_ is equal to:

COD_F_ = COD(*t* = *T*) − COD(*t* = 0) (8)

Since the permeate stream was completely returned back to the reactor, this quantity of COD not found any more in the feedstock is equal to the COD which holds on the membrane forming the fouling layer. In Table 2, the COD data at *t* = 0 and *t* = *T* are reported, and it is possible to notice that smaller particles appears to give rise to an increased value of COD_F_, that is a higher amount of accumulation on the membrane if compared to bigger ones. This result may be justified by the reduction of the apparent pore size, capable of rejecting more particles and thus accumulating them over the surface of the membrane. As a consequence, the increase of the COD rejection value can be attributed by both the reduction of the apparent pore size and the use of organic matter to build a more concentrated fouling layer.

The main problem in using Equation (7) is that the value of k is unknown and not measurable. In order to overcome the limitation, the ratio of permeate fluxes β was evaluated as:

β = *J*_p_|_UFNF_·*J*_p_^−1^|_ONF_ (9)

by adopting the Cozeny-Carman Equation:

*J*_p_ (L·h^−1^·m^−2^) = 180^−1^TMP·*d*_f_^2^·ε_f_^3^·(1 − ε_f_)^−2^·µ^−1^·*x*^−1^ (10)

and finally, by merging Equation (5) to Equation (9) together:

β = [*d*_f_^2^·*m*_f_^−1^·ε_f_^3^·(1 − ε_f_)^−2^·*D*_f_]|_UFNF_·[*d*_f_^2^·*m*_f_^−1^·ε_f_^3^·(1 − ε_f_)^−2^·*D*_f_]^−1^|_ONF_ (11)

The input data, reported in Table 3, allowed the solving of Equation (11) and a value of β equal to 1.06 was obtained.

The difference between the calculated value of β, (also considering all the strict hypothesis assumed) and the experimental one, equal to 1.14, is approximately 8%, which can be considered satisfying to justify the observed NF membrane behavior by the proposed theoretical construction in this case study. Although other systems may not follow the model, the basic mechanisms should hold on and it appears that in certain circumstances, depending of the feedstock in contact with the membrane, the fouling layer forming on top of membrane surfaces may exhibit a lower apparent pore size if compared to that of the membrane, and, as a consequence, may lead to a sensible increase of the selectivity.

## 4. Conclusions

In this work, a nanofiltration membrane was used to replace the secondary clarifier in a SSR activated sludge system for the treatment of a civil wastewater. It was observed that membrane selectivity towards the separation of organic matter strongly increased when an ultrafiltration pretreatment step was adopted, thus achieving rejection values of COD up to 76%.

This different behavior was attributed to the formation of a selective secondary membrane, consisting in the fouling layer on top of the membrane surface. A simple model was developed and proposed in this work, exhibiting satisfying accordance with the experimental data obtained in a case study.

Irreversible fouling of membranes should always be avoided in order to guarantee the longevity of the membrane modules. On the other hand, fouling at sub boundary flux conditions may be profitable to obtain selectivity values not reachable by other means. In this case, the fouling layer may improve the system’s performances without significantly affecting productivity and longevity.
